# Determination of anthracnose (*Colletotrichum fructicola*) resistance mechanism using transcriptome analysis of resistant and susceptible pear (*Pyrus pyrifolia*)

**DOI:** 10.1186/s12870-024-05077-6

**Published:** 2024-06-28

**Authors:** Xiaomei Tang, Fen Lu, Ziwen Xiao, Yue Wang, Guoqing Hu, Kexin Cai, Ruichang Yin, Wei Song, Luoluo Xie, Guoling Guo, Wenming Wang, Lun Liu, Li Liu, Zhenfeng Ye, Wei Heng, Xianping Guo, Dongsheng Wang, Bing Jia

**Affiliations:** 1 Anhui Province Key Laboratory of Horticultural Crop Quality Biology, School of Horticulture, Anhui Agricultural University, Hefei, 230036 China; 2 Sation of Science and Technology of Shexian, Huangshan, Anhui Province China; 3 Institute of Horticulture, Henan Academy of Agricultural Sciences, Zhengzhou, Henan Province 450002 China

**Keywords:** Pear, *Colletotrichum fructicola*, Disease resistance, RNA sequencing, MAPK signaling, Calcium signaling

## Abstract

**Background:**

Anthracnose, mainly caused by *Colletotrichum fructicola*, leads to severe losses in pear production. However, there is limited information available regarding the molecular response to anthracnose in pears.

**Results:**

In this study, the anthracnose-resistant variety ‘Seli’ and susceptible pear cultivar ‘Cuiguan’ were subjected to transcriptome analysis following *C. fructicola* inoculation at 6 and 24 h using RNA sequencing. A total of 3186 differentially expressed genes were detected in ‘Seli’ and ‘Cuiguan’ using Illumina sequencing technology. Gene Ontology and Kyoto Encyclopedia of Genes and Genomes pathway analyses indicated that the transcriptional response of pears to *C. fructicola* infection included responses to reactive oxygen species, phytohormone signaling, phenylpropanoid biosynthesis, and secondary metabolite biosynthetic processes. Moreover, the mitogen-activated protein kinase (MAPK) signaling pathway and phenylpropanoid biosynthesis were involved in the defense of ‘Seli’. Furthermore, the gene coexpression network data showed that genes related to plant–pathogen interactions were associated with *C. fructicola* resistance in ‘Seli’ at the early stage.

**Conclusion:**

Our results showed that the activation of specific genes in MAPK, calcium signaling pathways and phenylpropanoid biosynthesis was highly related to *C. fructicola* resistance in ‘Seli’ and providing several potential candidate genes for breeding anthracnose-resistant pear varieties.

**Supplementary Information:**

The online version contains supplementary material available at 10.1186/s12870-024-05077-6.

## Introduction

Pear (*Pyrus pyrifolia*) belongs to the *Pyrus* genus in the *Rosaceae* family and is known for its nutritional and health benefits. In the development of the pear industry, the prevalence of pathogens and insects is becoming a major concern. Pear anthracnose, caused by *Colletotrichum* spp., is one of the most devastating fungal diseases in the pear industry. It causes severe necrosis symptoms on leaves and fruits, accelerating the dropping of pear fruits and leaves, resulting in considerable yield losses [1–3]. *Colletotrichum* spp. consists of different strains, with *Colletotrichum fructicola* being the predominant pathogenic species that causes pear anthracnose in China [3]. The control of anthracnose mainly relies on the use of flusilazole, difenoconazole, and pyraclostrobin, which results in the soil and water pollution with long-term usage [2]. One of the environmentally and economically friendly approaches is to improve the resistance of host plants.

Host plants have evolved complex resistance mechanisms during their interactions with pathogens [4]. The first line of plant defense is to activate pathogen/microbe-associated molecular patterns (PAMPs/MAMPs) triggered immunity (PTI) through cell surface transmembrane recognition receptors (PRRs), including the mitogen-activated protein kinase (MAPK) pathway [5–8]. After pathogen recognition, a range of resistance responses are elicited, such as calcium influx pathways, MAPK pathway signaling, phytohormone signaling pathways, generation of reactive oxygen species (ROS) and production of antimicrobial compounds [9, 10]. However, for successful invasion, pathogens secrete numerous virulence proteins (effectors) to suppress PTI. In turn, plants have evolved resistance genes that can specifically recognize effectors; this recognition activates the second line of plant immune response known as effector-triggered immunity (ETI) to further restrict pathogen growth [6, 11]. During this process, plants usually produce ROS and elicit a hypersensitive response (HR), which eventually results in host programmed cell death (PCD) [7, 12]. Plant hormones, including salicylic acid (SA), jasmonic acid (JA), ethylene (ET), cytokinins (CKs), abscisic acid (ABA), brassinosteroids (BR), and gibberellin (GA), play synergistic or antagonistic roles in the plant defense system [13, 14].

Understanding the mechanism by which *Colletotrichum* species interact with plants is crucial for anthracnose control. Comparative transcriptome sequencing has been widely used to determine the mechanisms underlying plant–pathogen interactions, and many studies have investigated the mechanism of anthracnose resistance. For example, the MAPK-mediated activation of resistance genes involved in HR and H_2_O_2_ accumulation is a critical mechanism in tea plants in response to *Colletotrichum fructicola* infection, which was identified using comparative transcriptome sequencing of the susceptible tea cultivar ‘Longjing 43’ and resistant cultivar ‘Zhongcha 108’ [15]. In soybean, the resistance of ‘Zhechun No. 2’ to *C. truncatum* was related to JA, auxin, MAPK, calcium signaling, resistance gene expression, and terpenoid metabolism [16].

Despite some studies about the interactions of plants and *Colletotrichum* species, the complex molecular mechanisms underlying the interactions between pear and *C. fructicola* remain poorly understood. Previous research on ‘Cuiguan’ transcriptome after infection with two type *Colletotrichum fructicola* strain (PAFQ31 and PAFQ32) has been revealed different defense response. For example, transcriptome data of ‘Cuiguan’ after infection with highly pathogenic strain PAFQ31 (causing severe early leaves defoliation), the differently expressed genes were related with jasmonic acid (JA), ethylene (ET), and abscisic acid (ABA) signaling pathways, especially the increased endogenous JA levels, while the differently expressed genes in ‘Cuiguan’ after infection with weak pathogenic strain PAFQ32 were mainly related with salicylic acid (SA) signaling pathway [17]. Another recent study revealed that the resistance to early pear defoliate disease was associated with the expression levels of *WRKY* and *ERF* transcription factors [18].

To better understand the mechanism of pear anthracnose resistance, we performed transcriptome analysis of the anthracnose-resistant variety ‘Seli’ and highly susceptible variety ‘Cuiguan’ after infection with *C. fructicola* and sterile water at 6 and 24 h using RNA sequencing (RNA-seq). Our study revealed that the plant hormones, phenylpropanoid, and activation of PTI and ETI were related with the defense response to *C. fructicola* infection in ‘Seli’ and ‘Cuiguan’ leaves. What’s more, that the activation of specific genes in MAPK, calcium signaling pathways, and phenylpropanoid biosynthesis was highly related with *C. fructicola* resistance in ‘Seli’. Furthermore, we identified several potential candidate resistant genes, and provied new information about the mechanism of pear anthracnose resistance.

## Results

### *P. pyrifolia* ‘Seli’ exhibits higher resistance to *C. fructicola* than ‘Cuiguan’

**Fig. 1:**
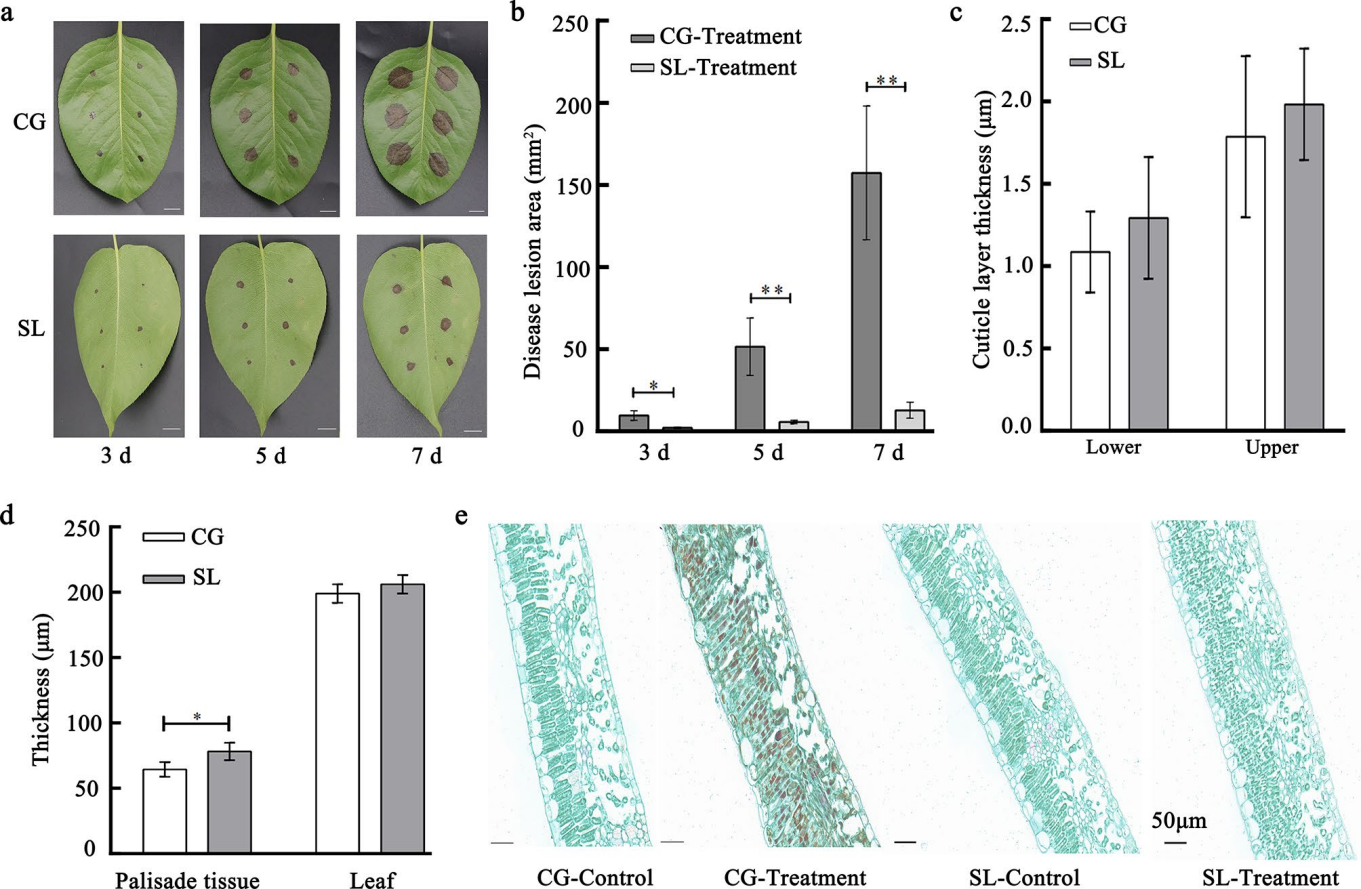
Pear varieties of ‘Seli’ (SL) and ‘Cuiguan’ (CG) were inoculated with *C. fructicola* (1 × 10^8^ CFU/mL). **(a)** Phenotypes of ‘Seli’ and ‘Cuiguan’ leaves at 3, 5, and 7 days after inoculation. Scale bar = 1 cm. **(b)** Disease lesion area of ‘Seli’ and ‘Cuiguan’ leaves at 3, 5, and 7 days after inoculation. **(c)** Cuticle layer thickness (average of thirty independent replicates) of ‘Cuiguan’ and ‘Seli’. (d) Palisade tissue (average of thirty independent replicates) and leaf thickness (average of twenty independent replicates) of ‘Cuiguan’ and ‘Seli’. Data from thirty or twenty independent replicates are expressed as the mean ± standard deviation. Different letters above the bars represent significant differences according to Duncan’s multiple range test (*P* = 0.05; *n* = 30). **(e)** Leaf internal anatomy structure at 0 and 5 days after inoculation. Scale bar = 50 μm

Leaves of ‘Seli’ and ‘Cuiguan’ were inoculated with *C. fructicola* to evaluate their disease resistance. Disease symptoms appeared in both varieties after 3 days of inoculation. However, the lesion area of ‘Seli’ was significantly smaller than that of ‘Cuiguan’ at 3, 5, and 7 days after inoculation (Fig. 1a and b). To explore the difference of leaf surface texture in the two varieties, we measured cuticle layer, palisade tissue, and leaf thickness by CaseViewer software (Fig. 1c and d). Our results illustrated that lower / upper cuticle layer, and leaf thickness was no significant difference in ‘Seli’ and ‘Cuiguan’, while the palisade tissue in ‘Seli’ was notably higher than in ‘Cuiguan’ (Fig. 1c and d). The paraffin section of inoculated leaves revealed that the palisade and spongy tissues of ‘Cuiguan’ leaves were severely damaged, whereas those of ‘Seli’ leaves remained largely unaffected (Fig. 1e).

### Enzyme activity analysis

**Fig. 2:**
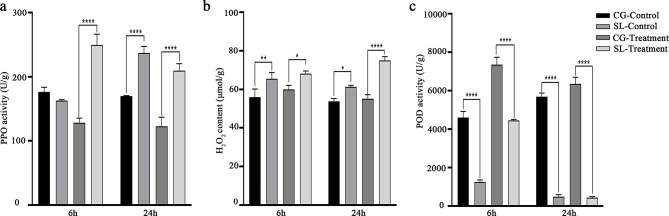
Enzyme activity of ‘Seli’ (SL) and ‘Cuiguan’ (CG) leaves after inoculation with *C. fructicola* (treatment) and sterile water (control). **(a-c)** The leaf enzyme activity of polyphenol oxidase (PPO), H_2_O_2_ content, and peroxidase (POD) at 0, 6 and 24 h after inoculation. Data from three independent replicates are expressed as the mean ± standard deviation. Different letters above the bars represent significant differences according to Duncan’s multiple range test (*P* = 0.05; *n* = 3)

To elucidate the early physiological response, the ‘Seli’ and ‘Cuiguan’ samples were collected after inoculation with *C. fructicola* at 6, and 24 h and analyzed. The enzyme activity illustrated that the polyphenol oxidase (PPO) activity, and H_2_O_2_ content in ‘Seli’ leaves were notably higher than those in ‘Cuiguan’ leaves at both 6 and 24 h after inoculation (Fig. 2a and b). Furthermore, peroxidase (POD) activity was remarkably lower in ‘Seli’ leaves than in ‘Cuiguan’ leaves at 6 and 24 h after inoculation (Fig. 2c). The different effect between ‘Seli’ and ‘Cuiguan’ in response to *C. fructicola* infection may be involved in anti-oxidation.

### Differentially expressed genes in ‘Seli’ and ‘Cuiguan’ in response to ***C. fructicola*** infection

**Fig. 3:**
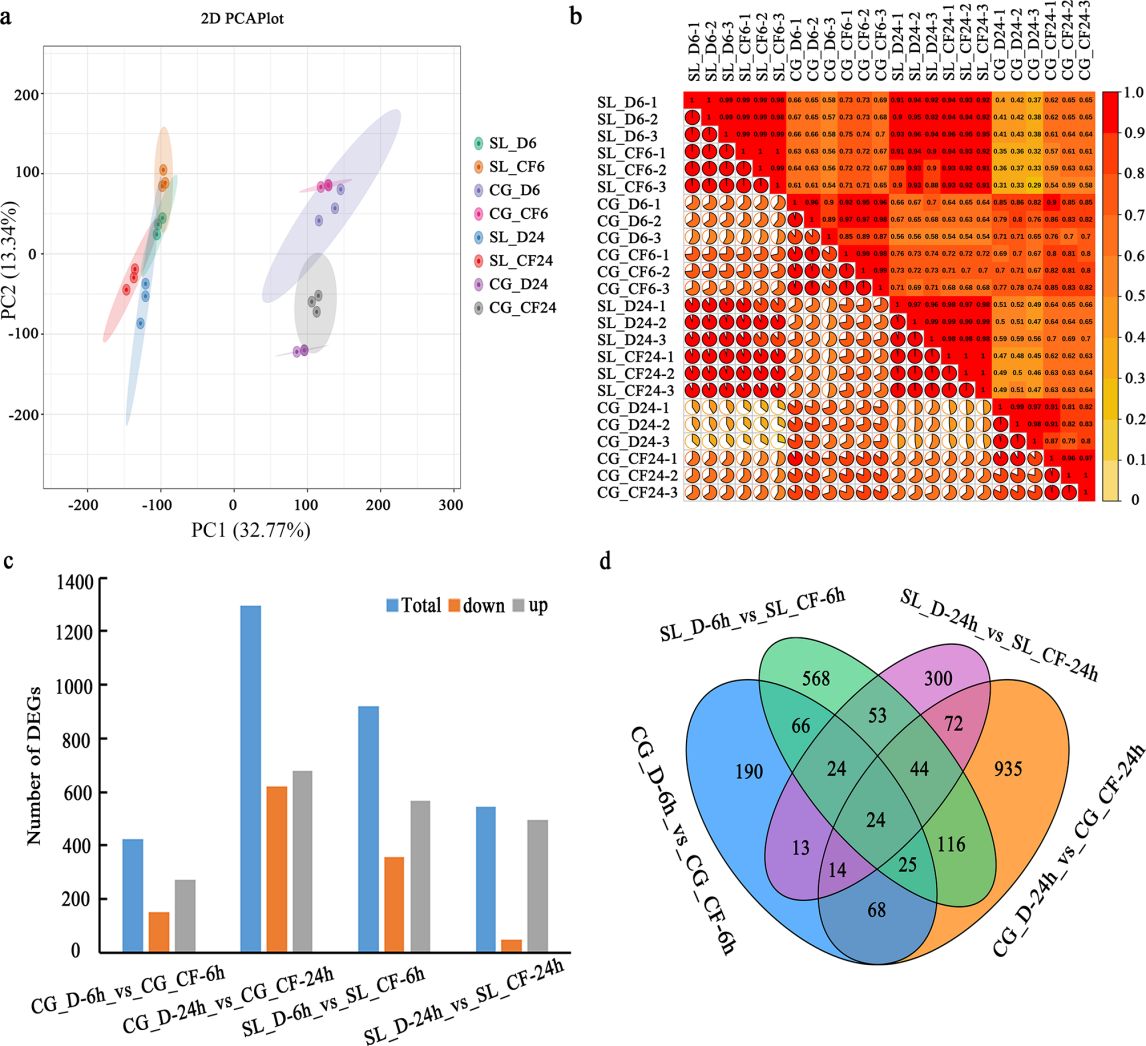
Statistical analysis of differentially expressed genes (DEGs) in response to *C. fructicola* inoculation at 6 and 24 h. **(a)** Principal component 1 (32.77% of the variance) and principal component 2 (13.34% of the variance). **(b)** Pearson’s correlation coefficient testing in ‘Seli’ and ‘Cuiguan’ sample datasets. Pie chart area represents the size of the correlation coefficient r; Diagonal of the upper right corner of the grid for the corresponding sample pearson correlation coefficients r. The deeper the red color corresponding squares, the stronger the correlation between two samples. **(c)** The overall upregulated and downregulated DEGs in ‘Seli’ and ‘Cuiguan’ post-infection. **(d)** Venn diagram showing the transcript distribution in ‘Seli’ and ‘Cuiguan’ samples inoculated with *C. fructicola* at 6 and 24 h. SL_CF and CG_CF indicated that ‘Seli’ and ‘Cuiguan’ leaves were inoculated with *C. fructicola*, respectively, and SL_D and CG_D indicated that ‘Seli’ and ‘Cuiguan’ leaves were inoculated with sterile water, respectively. Duration (hours) of *C. fructicola* inoculation is indicated by the number following ‘Seli’ or ‘CuiGuan’ names, e.g., SL_CF-6 h indicates 6 h of *C. fructicola* inoculation in ‘Seli’, whereas SL_D-6 h indicates 6 h of sterile water inoculation in ‘Seli’

To determine the molecular basis for ‘Seli’ and ‘Cuiguan’ in response to *C. fructicola*, A total of 24 samples were collected from ‘Seli’ and ‘Cuiguan’ leaves inoculated with *C. fructicola* (treatment) and sterile water (control) at 6 and 24 h, respectively. Each variety including 12 samples, and each sample contains three biological replicates. The 24 sample libraries were constructed, each with 40–55 million reads. The GC content and the number of repeat sequence reads were calculated using FastQC software (Table S1 in Additional File 1). More than 90% of the clean reads were mapped to the reference genome of *P. pyrifolia* [19], thus indicating that the quality of the transcriptome sequencing data was reliable. Principal component analysis (PCA) results showed that samples from the treatment of ‘Seli’, ‘Cuiguan’ and respective control groups were clearly divided into two categories, as well as the differentiating between ‘Seli’ and ‘Cuiguan’. The first two principal components (PC1 and PC2) explain 32.77% and 13.34% of the variation, respectively (Fig. 3a). Additionally, it is possible to observe a high reproducibility (*r* > 0.89) among biological replicates in ‘Seli’ and ‘Cuiguan’ treatment and control groups. (Fig. 3b). The total mapped reads of all genes were used for differential expression analysis using DESeq |log2(fold change)| ≥1 and false discovery rate FDR ≤ 0.05. A total of 3186 differentially expressed genes (DEGs) were identified in ‘Seli’ and ‘Cuiguan’ at 6 and 24 h after inoculation (Fig. 3c). Comparative analysis of DEGs between the two *C. fructicola* infection time points in ‘Seli’ revealed that they shared 145 DEGs (i.e. SL_6 and SL_24) (Fig. 3d). A similar comparison for ‘Cuiguan’ revealed 131 common genes at the two time points (Fig. 3d). In addition, the DEGs were analyzed between ‘Seli’ and ‘Cuiguan’ at 6 and 24 h post-infection, and we found that only 24 DEGs overlapped across all treatment samples (Fig. 3d).

### Functional annotation of DEGs and pathway enrichment analysis

**Fig. 4:**
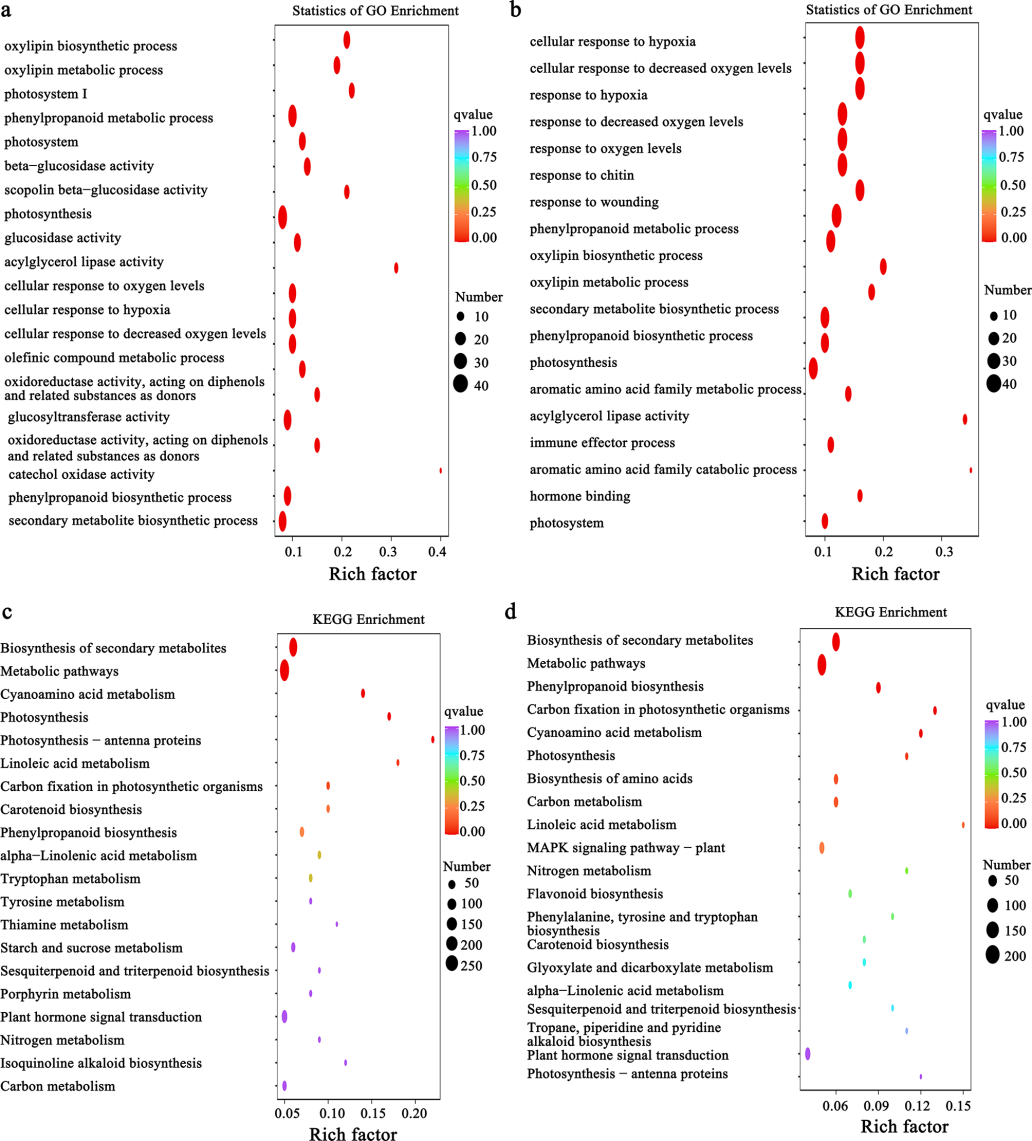
Gene Ontology (GO) and Kyoto Encyclopedia of Genes and Genomes (KEGG) enrichment pathways of differentially expressed genes (DEGs) in ‘Seli’ (SL) and ‘Cuiguan’ (CG) at 6 and 24 h after inoculation with *C. fructicola*. **(a)** GO enrichment of ‘Cuiguan’ treatment versus ‘Cuiguan’ control. **(b)** GO enrichment of ‘Seli’ treatment versus ‘Seli’ control. **(c)** KEGG enrichment of ‘Cuiguan’ treatment versus ‘Cuiguan’ control. **(d)** KEGG enrichment of ‘Seli’ treatment versus ‘Seli’ control. Treatment indicates ‘Cuiguan’ and ‘Seli’ were inoculated with *Colletotrichum fructicola*, and control indicates ‘Cuiguan’ and ‘Seli’ were inoculated with sterile water. The q-value is an adjusted p-value ranging from 0 to 1, and a lower value indicates greater pathway enrichment

To fully understand the biological process of the identified DEGs in ‘Seli’ and ‘Cuiguan’ at 6 and 24 h after *C. fructicola* infection, Gene Ontology (GO) and Kyoto Encyclopedia of Genes and Genomes (KEGG) enrichment analyses were performed. GO enrichment analysis of the DEGs revealed that there were six common pathways in the comparisons of ‘Cuiguan’ treatment versus ‘Cuiguan’ control and ‘Seli’ treatment versus ‘Seli’ control, which included response to phenylpropanoid biosynthetic process, phenylpropanoid metabolic process, and secondary metabolite biosynthetic process, photosynthesis, response to oxygen levels, and decreased oxygen levels. In addition, the DEGs of ‘Seli’ treatment versus ‘Seli’ control were enriched in response to chitin, immune effector, and response to wound (Fig. 4a and b). The KEGG enrichment results revealed that most DEGs of ‘Cuiguan’ treatment versus ‘Cuiguan’ control and ‘Seli’ treatment versus ‘Seli’ control were enriched in the biosynthesis of secondary metabolites, metabolic pathways and plant hormone signal transduction. However, according to the enrichment pathway results, MAPK signaling, and flavonoid biosynthesis pathways were uniquely enriched in ‘Seli’ treatment versus ‘Seli’ control (Fig. 4c and d). We speculated that the DEGs involved in the pathways of secondary metabolites, biosynthetic processes, and MAPK signaling pathway are closely related to ‘Seli’ resistance to *C. fructicola* infection.

**Fig. 5:**
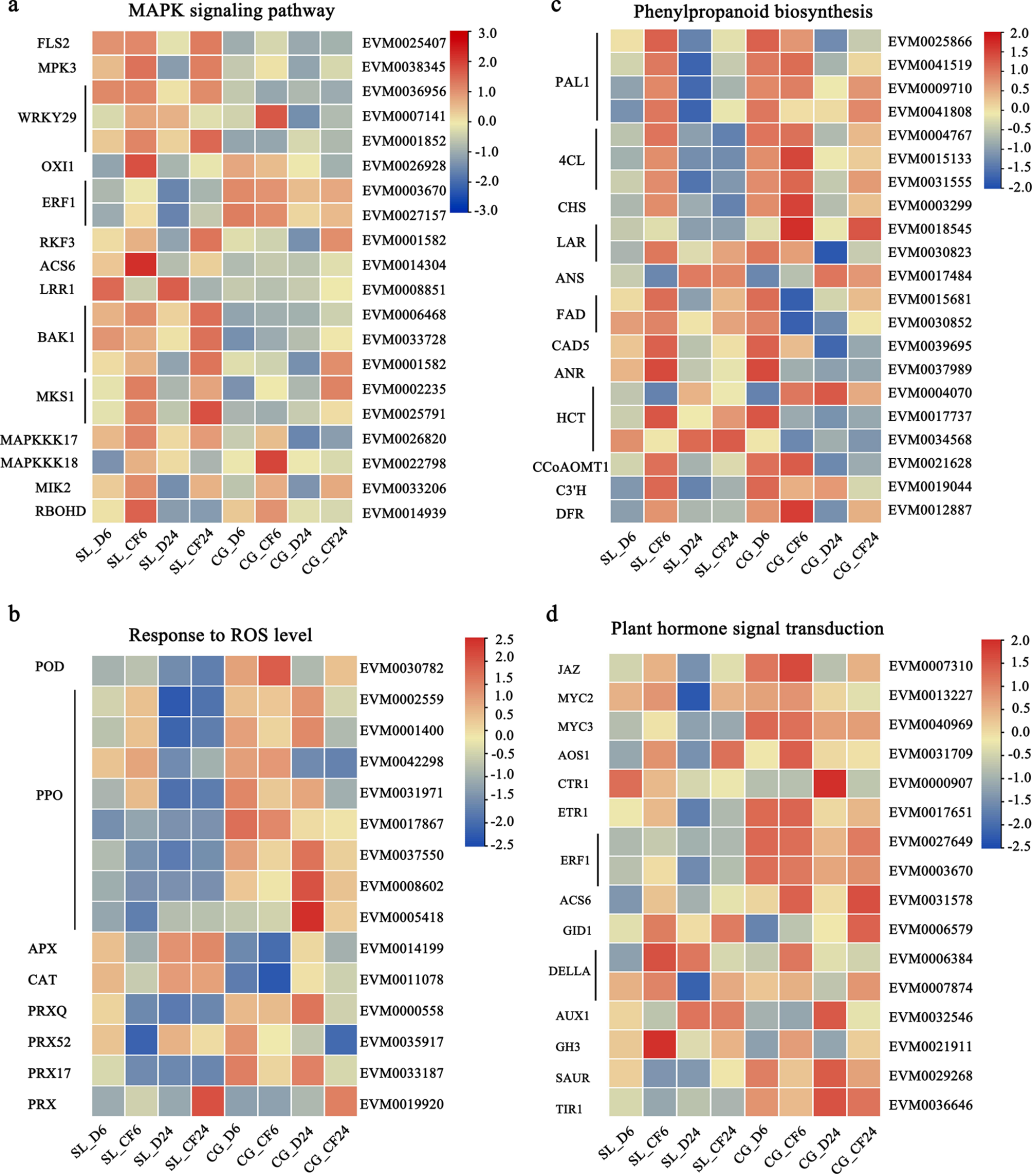
Heatmap analyses of the differentially expressed genes (DEGs) linked to disease resistance in ‘Seli’ (SL) and ‘Cuiguan’ (CG) leaves after *C. fructicola* infection at 6 and 24 h. **(a)** Heatmap of DEGs involved in the MAPK signaling pathway. **(b)** Heatmap of genes response to ROS level. **(c)** Heatmap of DEGs involved in phenylpropanoid biosynthesis. **(d)** Heatmap of DEGs involved in plant hormone signal transduction. Treatment: D, sterile water; CF, *C. fructicola*

### Genes potentially involved in ‘Seli’ resistance to *C. fructicola* infection

We further analyzed the gene expression levels in three KEGG pathways, and one GO enrichment pathway: MAPK signaling pathway, phenylpropanoid biosynthesis, plant hormone signal transduction, and response to ROS level. The plant MAPK signaling pathway plays a pivotal role in plant disease resistance [15, 20]. Herein, we revealed that genes related to the PTI pathway were induced in ‘Seli’, including leucine-rich repeat (LRR) receptor-like kinase (*FLS2*), BRI1-associated receptor kinase 1 (*BAK1*), *MPK3*, WRKY-type transcription factor 29 (*WRKY29*), oxidative signal inducible 1 (*OXI1*), MAP kinase substrate 1 (*MKS1*), and NADPH oxidase (*RBOHD*) (Fig. 5a). We inferred that the expression of these early defense genes in ‘Seli’ could induce ROS accumulation and cell death, thus restricting *C. fructicola* expansion in the early stage.

Previous studies have been reported that antioxidative enzymes genes of Class III peroxidases (*POD*), catalase activity (*CAT*), ascorbate peroxidase (*APX*) and peroxidases (*PRX*) were involved in scavenging H_2_O_2_ [21]. In this study, we found that the expression level of *POD* was higher in CG_CF than in CG_D at 6 and 24 h. While the expression of *APX*, *CAT*, and *PRX52* was notably downregulated in ‘Seli’ after *C. fructicola* infection at 6 h (Fig. 5b). Intriguingly, two of *PPO* genes (EVM0001400, and EVM0031971) expression was notably upregulated in ‘Seli’ after *C. fructicola* infection at 6 h, while the two genes expression was remarkably downregulated in ‘Cuiguan’ after *C. fructicola* infection at 24 h. What’s more, except for *PPOs* (EVM0042298 and EVM0017867), other four *PPO* gene expressions were remarkably downregulated in ‘Cuiguan’ after *C. fructicola* infection at 24 h (Fig. 5b). The difference of the enzyme acivity in ‘Seli’ and ‘Cuiguan’ in response to *C. fructicola* infection may be related with the differential expression of antioxidative enzymes related genes.

Phenylpropanoid biosynthesis is an important secondary metabolism pathway in plant disease resistance [20]. We found that the expression of genes related to flavonoid biosynthesis, including 4-coumarate: coenzyme A ligase (*4CL*), phenylalanine ammonia lyase (*PAL*), and caffeoyl-CoA O-methyltransferase 1 (*CCoAOMT1*) was remarkably upregulated in ‘Seli’ and ‘Cuiguan’ leaves after *C. fructicola* infection. However, the expression of genes related to fatty acid and flavonoid biosynthesis, including fatty acid desaturase, cinnamon alcohol dehydrogenase 5 (*CAD5*), anthocyanidin reductase (*ANR*), chalcone synthase (*CHS*), p-coumarate 3-hydroxylase (*C3’H*) and two shikimate hydroxycinnamoyl transferases (*HCT*), and Leucoanthocyanidin reductase (*LAR*, EVM0030823) was substantially upregulated in ‘Seli’ when inoculated with *C. fructicola* at 6 h (Fig. 5c).

Genes involved in different plant hormone signal transduction pathways were also characterized. We found that transcriptional repressors such as jasmonate zim-domain (*JAZ*) in JA signaling as well as SCF-type E3 ligase complex (*GID1*) in the GA signaling pathway were upregulated in ‘Cuiguan’ and ‘Seli’ after inoculation with *C. fructicola*, while *DELLA* was uniquely upregulated in ‘Seli’ after inoculation (Fig. 5d). AOS is the second enzyme in the biosynthesis of the plant defensive hormone JA [22]. We found that the expression of *AOS1* was upregulated in ‘Seli’ and ‘Cuiguan’ at 6 h (Fig. 5d). Meanwhile, we found that ethylene and auxin synthesis genes including ethylene response factor 1 (*ERF1*), ACC synthase 6 (*ACS6*), auxin-influx carrier (*AUX1*) and Gretchen Hagen 3 (*GH3*) were notably upregulated in ‘Cuiguan’ and ‘Seli’ after inoculation with *C. fructicola.* However, ethylene receptor *ETR1* and small auxin-up RNA (*SAUR)* gene expression was substantially upregulated in ‘Seli’ treatment.

### Weighted correlation network analysis (WGCNA) for the DEGs of ‘ Seli’ and ‘ Cuiguan’ in response to *C. fructicola* infection

**Fig. 6:**
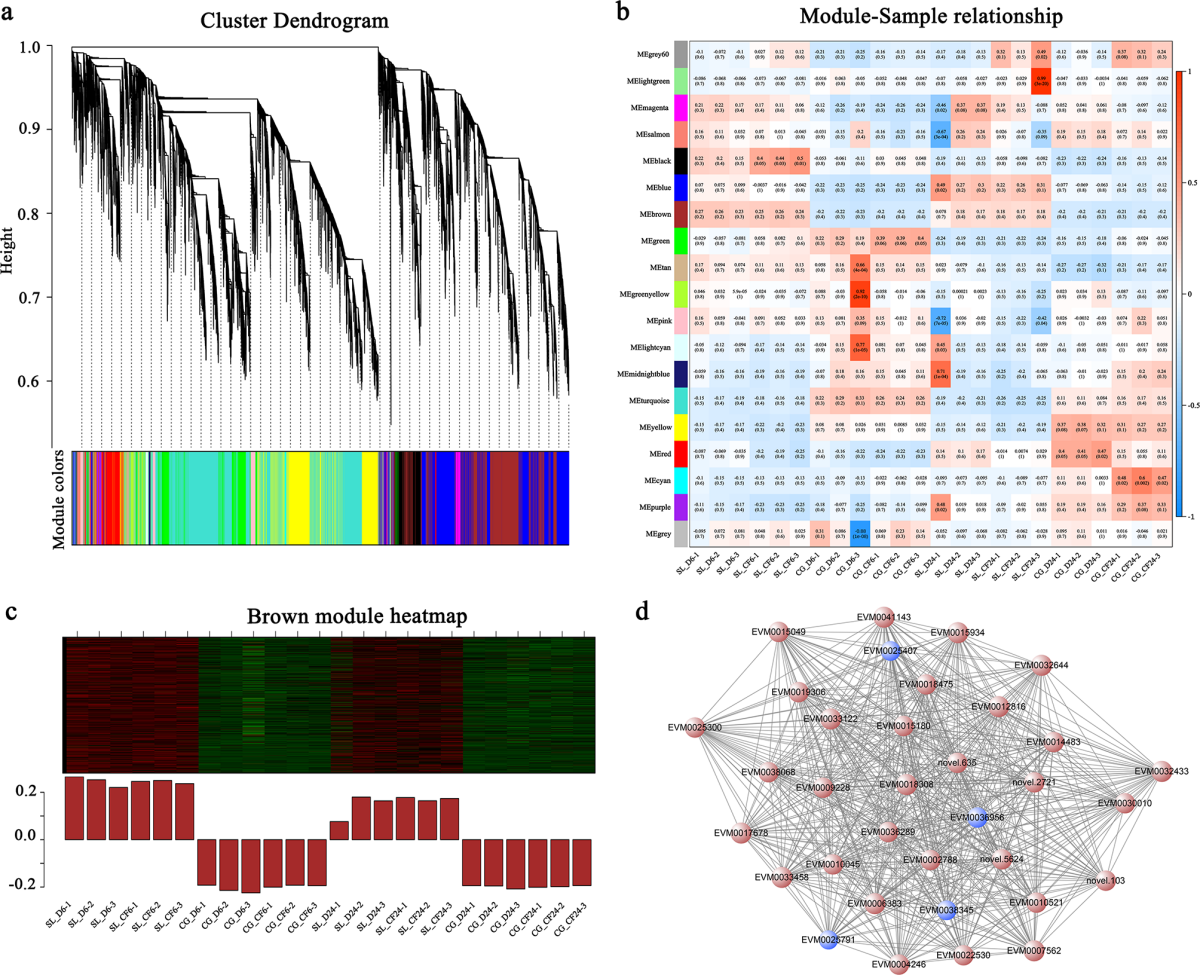
Weighted gene coexpression network. **(a)** Dendrogram clusters showing co-expression modules identified by weighted correlation network analysis (WGCNA) of gene expression and samples. The major tree branches constitute 19 modules. Different modules are indicated by different colors. **(b)** Heat map showing correlation between the samples and modules. The correlation coefficient (top) and P-value (bottom) are indicated in each rectangle. The colored bar represents the correlation level between the samples and modules. **(c)** Representative modules identified in the heatmap. **(d)** Protein–protein interaction network based on the hub genes (blue nodes) from the brown module

To determine the gene regulatory network of ‘Seli’ and ‘Cuiguan’ in response to *C. fructicola* infection, a weighted gene coexpression network was constructed based on 3186 DEGs identified in the RNA-seq data. In total, the network was divided into 19 modules according to the correlations between the modules and samples (Fig. 6a). The heatmap of module-sample cor-relations indicated that the accumulation of transcripts for the brown module was highly correlated with ‘Seli’ and ‘Cuiguan’ sample (Fig. 6b). Then, the expression of the genes in the brown module was analyzed, the results illustrated that these DEGs was upregulated in ‘Seli’ and downregulated in ‘Cuiguan’ (Fig. 6c). Furthermore, we analyzed the biological processes of the DEGs in the brown module and found that these DEGs were enriched in the plant–pathogen interaction and MAPK signaling pathway (Fig. S1 in Additional File 2). We further revealed that these DEGs were highly related to *C. fructicola* infection. Combining the KEGG enrichment results and gene function prediction, *FLS2*, *MPK3*, *MKS1*, and *WRKY29* in the brown module appeared to be the hub genes that play important roles in plant–pathogen interaction (Fig. 6d).

### Validation of RNA-seq data via quantitative reverse transcription polymerase chain reaction (qRT–PCR)

**Fig. 7:**
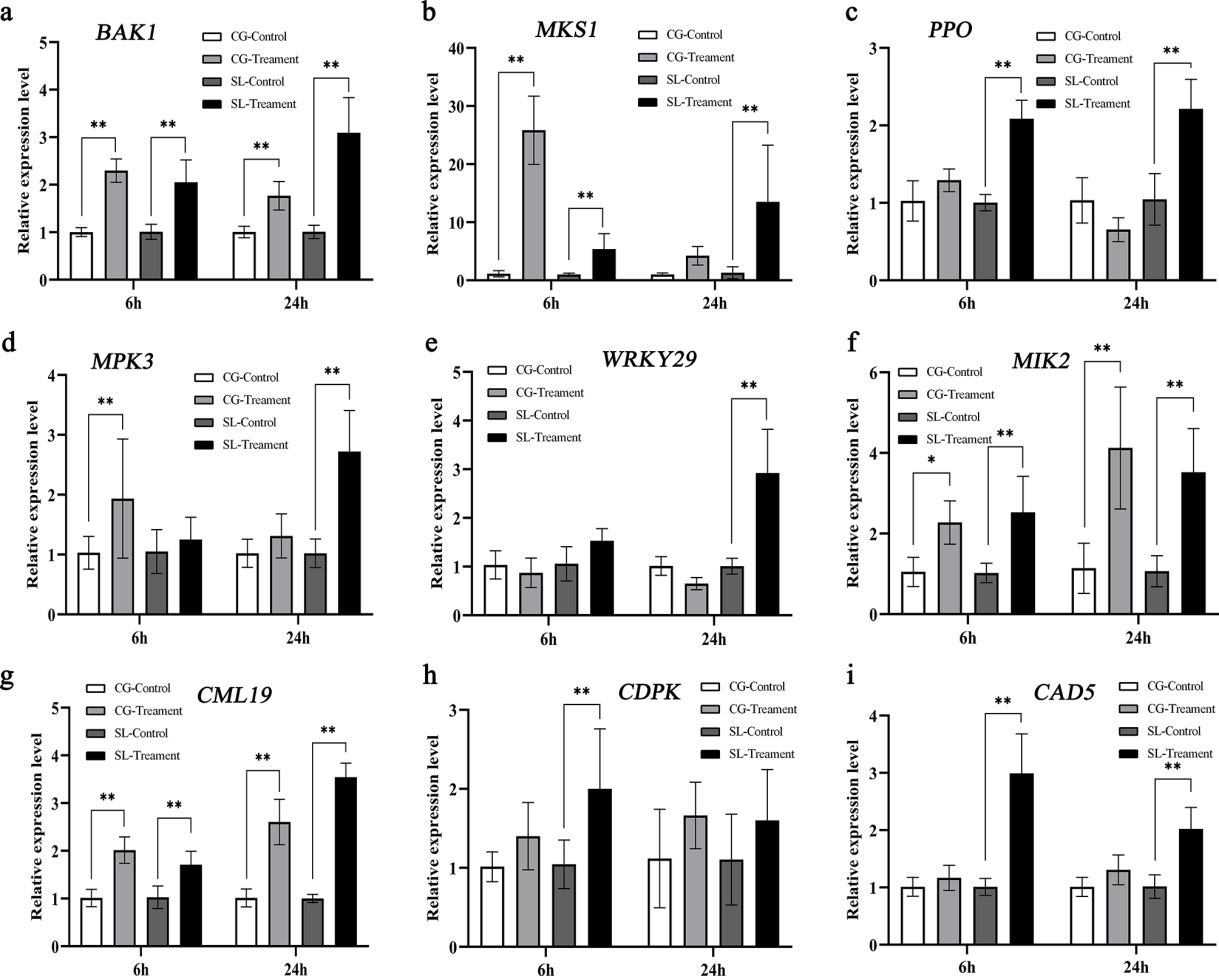
qRT–PCR validation of the selected differentially expressed genes (DEGs) in ‘Seli’ and ‘Cuiguan’ leaves after C. fructicola inoculation. (a-f) Nine genes were evaluated: BRI1-associated receptor kinase 1 (BAK1), MAP kinase substrate 1 (MKS1), polyphenol oxidase (PPO), mitogen-activated protein kinase 3 (MPK3), WRKY-type transcription factor 29 (WRKY29), male discoverer 1-interacting receptor-like kinase 2 (MIK2), calmodulin-like protein 19 (CML19), calcium-dependent protein kinase (CDPK), and cinnamyl alcohol dehydrogenase 5 (CAD5). The treatment group was inoculated with C. fructicola, and the control group was inoculated with sterile water at 6 and 24 h. Gene expression levels were normalized to respective control group of ‘Seli’ and ‘Cuiguan’ at 6 and 24 h post-inoculation. The relative expression level was calculated using the 2−ΔΔCt method with actin as the reference gene. The statistical analysis was carried out by GraphPad Prism 8 software. Error bars indicate the standard deviation of three independent repetitions.

To confirm the quality of the transcriptome data, qRT–PCR was conducted for the nine candidate genes involved in MAPK signaling pathway, response to ROS level, and phenylpropanoid metabolism was also validated via qRT–PCR (Fig. 7). Our results showed that *BAK1*, *MKS1* and *PPO* gene expression was sustainably upregulated in ‘Seli’ at 6 and 24 h after *C. fructicola* inoculation (Fig. 7a, b and c).What’s more, the expression of *MPK3* and *WRKY29* was notably upregulated in ‘Seli’ at 24 h after *C. fructicola* inoculation (Fig. 7d and e). Meanwhile, the expression of male discoverer 1-interacting receptor-like kinase 2 (*MIK2*) and calmodulin-like protein 19 (*CML19*) was upregulated in ‘Seli’ and ‘Cuiguan’ at 6 and 24 h after inoculation (Fig. 7f and g). Furthermore, the expression of calcium-dependent protein kinase (*CDPK*), and *CAD5* was induced and upregulated in ‘Seli’ leaves after *C. fructicola* inoculation (Fig. 7h and i). Thus, most of the selected genes showed similar expression patterns as those of the RNA-seq data (Fig. 5a, b and c, Table S2 in Additional File 1).

## Discussion

*C. fructicola* is a severe disease-causing pathogen in many economic fruits, such as mango, banana, apple, citrus, and pear [2, 23–26]. It has been reported that most *Pyrus* varieties are susceptible to anthracnose, and most of the resistant varieties are wild germplasm [27]. The molecular mechanisms of *C. fructicola* resistance in pear remain unclear. Shan et al. (2023) investigated early leaf defoliation in 155 pear varieties and reported 19 resistant varieties (*P. pyrifolia*). They employed comparative transcriptomics and revealed the response of the resistant variety ‘Whasan.’ However, studies on the early defense responses to anthracnose are limited. ‘Seli,’ an excellent wild pear variety with local characteristics, is grown in Shexian, Anhui Province, China. Pears have high medicinal value (‘Shexian chronicle’ in 1995), and the leaves were found to be highly resistant to *C. fructicola* in our study (Fig. 1a and b).

Herein, we identified the comprehensive resistance mechanisms of ‘Seli’ compared with the susceptible variety ‘Cuiguan’ using RNA-seq. DEG analyses further revealed that a higher number of upregulated genes than downregulated genes were identified in ‘Seli’ and ‘Cuiguan’ samples at 6 and 24 h. In particular, most DEGs were upregulated in ‘Seli’ (Fig. 3c). These results are supported by a previous report of resistant tea plants inoculated with *C. fructicola* [15, 28]. GO and KEGG enrichment analyses illustrated that MAPK signaling pathway, response to ROS, phenylpropanoid biosynthesis, flavonoid biosynthesis, and plant hormone signal transduction pathway play crucial roles in pear’s response to anthracnose resistance (Fig. 4).

ROS bursts is as one of the earliest responses to pathogen invasion, and many studies have reported that HR and ROS bursts are crucial for resistance to tea anthracnose [15], citrus bacterial canker [29], and rice blast disease [30]. In our study, we observed that a higher number of DEGs were enriched in ROS generation in ‘Seli’, and the H_2_O_2_ content and PPO activity was significantly higher in ‘Seli’ than in ‘Cuiguan’ after infection with *C. fructicola* (Fig. 2a and b). These results demonstrated that ROS is critical for pear anthracnose resistance, and ROS production may be regulated by PTI signaling. PTI is the first line of defense that inhibits many pathogens, including bacteria, fungi, oomycetes, and viruses [7]. FLS2, a well-characterized PRR, initiates immune signaling and induces an ROS burst through instantaneous heterodimerization with the immune kinase *BAK1* [31–34]. We revealed that the expression of two *BAK1* and one *FLS2* genes was notably upregulated in ‘Seli’ after inoculation with *C. fructicola*, whereas the expression in ‘Cuiguan’ showed no difference (Fig. 5a), suggesting a role of conferring resistance to the variety. A previous report showed that the conserved effector *NIS1* of *C. orbiculare* could target *BAK1* to suppress both cell death and ROS generation [35]. Similarly, we inferred that the lower expression of *BAK1* was related to anthracnose susceptibility in ‘Cuiguan’.

The MAPK cascade acts as one of the earliest signaling responses to bacterial and pathogenic infections and is located downstream of FLS2 [20, 36]. In this study, we found that the expression of *MPK3* was notably upregulated in ‘Seli’ at 24 h after inoculation with *C. fructicola*. Previous studies have reported that *MPK3* is essential for cotton resistance to whitefly, and silencing of *GhMPK3* results in the suppression of MPK–WRKY-mediated hormone pathways and enhancement of whitefly susceptibility [37]. *MPK3* and *MPK6* are positive regulators of *Arabidopsis* defense responses that control ET and JA biosynthesis [38, 39]. Herein, we found that the ET biosynthesis gene *WRKY29* was remarkably upregulated in ‘Seli’ at 24 h after infection with *C. fructicola* (Figs. 5a and 6e), we inferred that ET biosynthesis is crucial for cell death and pear anthracnose defense in the early stages. Interestingly, previous report illustrated that *MKS1* could activate SA-dependent resistance through interaction with *WRKY* transcription factors *WRKY25* and *WRKY33*, and the overexpression of *MKS1* remarkably improved the SA content [40]. Here we found that *MKS1* expression was sustainably upregulated in ‘Seli’ at 6 and 24 h after inoculation, this indicated that *MKS1* play a vital role in pear anthracnose resistance. Calcium, a second messenger, performs crucial roles in plant signaling conduction, including responses to various pathogen stressors [41–43]. Furthermore, we revealed that most of the calcium signaling-related genes, including *CDPK* and *CMLs*, were remarkably upregulated in ‘Seli’ after *C. fructicola* infection (Table S2 in Additional File 1, Fig. 7h).

Phenylpropanoid metabolism, which is derived from multiple pathways, including flavonoid, terpenoid, lignin, and anthocyanin pathways, plays key roles in plant disease resistance [44]. We revealed that the expression of *PAL1*, *4CL*, and *CCoAOMT1* in flavonoid metabolites was upregulated in the early stage of *C. fructicola* infection at 6 h (Fig. 5b). This was similar to a previous report on *Stylosanthes* plants wherein infection with *C. gloeosporioides* was managed by upregulating the levels of genes and compounds in the flavonoid biosynthesis pathway [45]. Furthermore, we revealed that *CAD5* and *C3’H* in lignin metabolites was substantial upregulation in ‘Seli’ after *C. fructicola* inoculation at 6 h (Fig. 5b). Regarding these genes, previous studies have revealed that *CAD5* positively regulated shoot blight resistance in *Bambusa pervariabilis* × *Dendrocalamopsis grandis* by regulating the synthesis of lignin and flavonoids [46], and *C3’H* was associated with the synthesis of lignin and the secondary metabolic cell wall [47]. We inferred that these genes in ‘Seli’ may provide mechanical strength to the cell wall to restrict *C. fructicola* expansion. Previous research has demonstrated that *bHLH137* promotes proanthocyanidin and anthocyanin biosynthesis by regulating the expression of *LAR2* and is involved in the response to *C. gloeosporioides* infection in grapes [48]. Our results revealed that *ANR*, *CHS* and *LAR* (EVM0030823) related to anthocyanidins synthesis were uniquely upregulated in ‘Seli’ after *C. fructicola* inoculation at 6 h (Fig. 5b). The function of anthocyanidins in response to *C. fructicola* infection requires further investigation.

## Conclusion

**Fig. 8:**
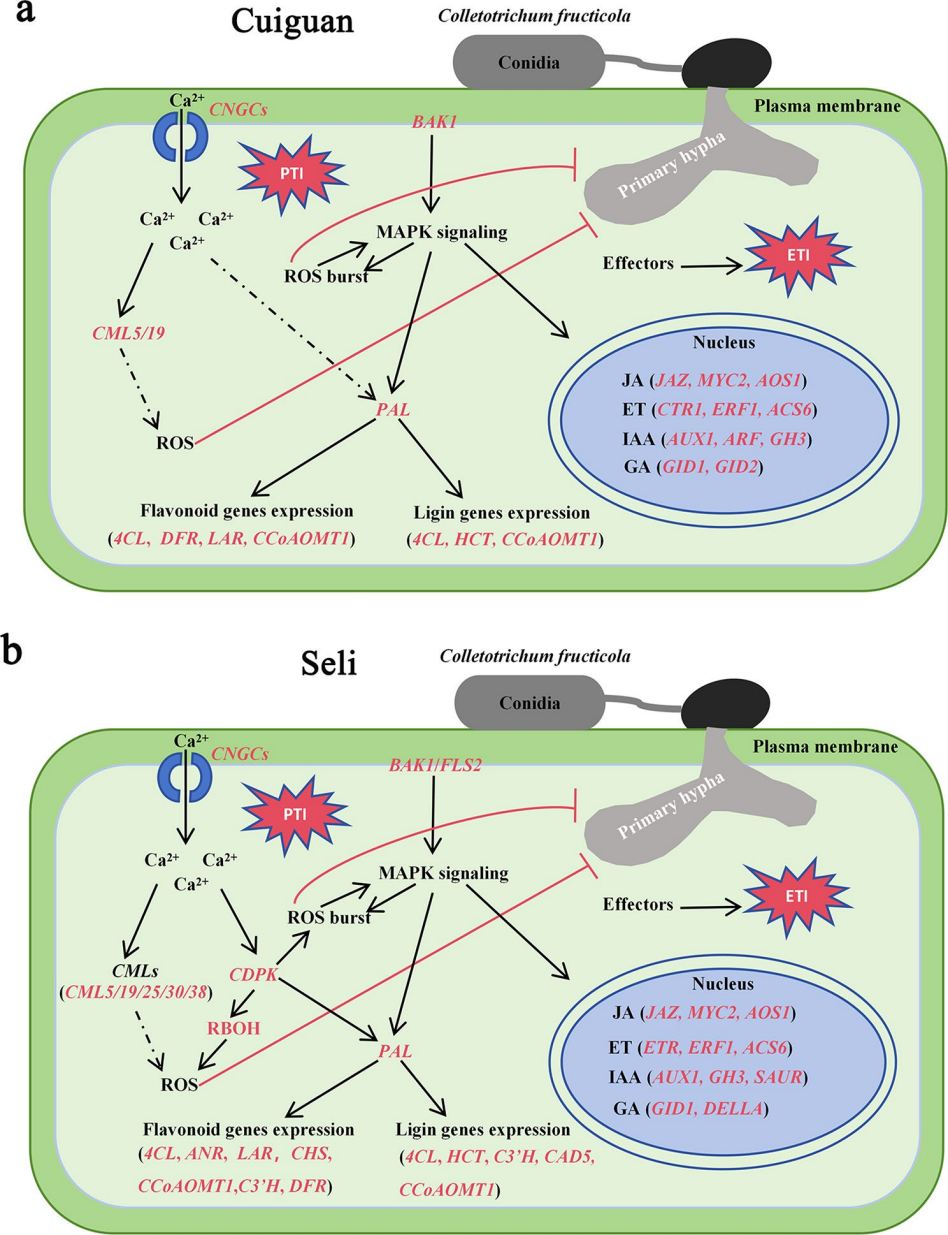
Hypothetical model for *P. pyrifolia* ‘Cuiguan’ and ‘Seli’ in response to *C. fructicola* infection **(a)** Defense response in ‘Cuiguan’ after infection with *C. fructicola.***(b)** Defense response in ‘Seli’ after infection with *C. fructicola.* The model was constructed based on the results of our study. Genes in red color were notably upregulated in ‘Cuiguan’ and ‘Seli’ after infection with *C. fructicola.* The morphological characteristics of *C. fructicola* conidia was exhibited as previous report [49]

Our study revealed that multiple biological processes, including response to ROS, plant hormones, phenylpropanoid, and activation of PTI and ETI, were stimulated in ‘Seli’ and ‘Cuiguan’ leaves in response to *C. fructicola* infection. Moreover, our results demonstrated that the activation of specific genes in MAPK (*RBOH*), calcium signaling pathways (*CDPK*, *CML25/30/38*), and flavnoid (*C3’H*, *CHS*, *ANR*) was highly related to *C. fructicola* resistance in ‘Seli’ (Fig. 8). These results provide new insights into the regulatory molecular mechanisms of pear anthracnose and highlight the importance of early defense response.

## Materials and methods

### Plant materials and pathogens

All experiments were performed with the permission of the relevant institution. Leaves of ‘Seli’ were obtained from the Shexian Shangfeng township, Huangshan City, Anhui Province, and Gangji Eco-agriculture Experimental Demonstration Base of Anhui Academy of Agricultural Sciences, Hefei City, Anhui Province, China. The ‘Cuiguan’ leaves were obtained from the germplasm garden of the Anhui Agricultural University (High Tech Agricultural Garden). *C. fructicola* was isolated from ‘Dangshansuli’ pear leaves and cultured on PDA medium for approximately 10 days at 25 °C for the production of conidia [1].

### Inoculation assay

Fully expanded pear leaves were punctured three holes with an inoculation needle (0.5 mm in diameter) on the left and right sides, and inoculated with 5 µL of *C. fructicola* conidia suspensions (1 × 10^8^ CFU/mL) (treatment) or sterile water (control), respectively. The disease lesion area (at least 36 inoculation sites on average) was measured using ImageJ 2.0 on days 3, 5 and 7 d.

### Pathological section observation and enzyme activity analysis

Tissue slices (5 × 5 mm) at the junction of healthy and diseased ‘Seli’ and ‘Cuiguan’ leaves were obtained on day 5, and the samples were fixed in 50% formaldehyde acetic acid (FAA) fixating solution for 36 h. The fixed tissues were sent to Seville Biotechnology (Wuhan, China) for paraffin section observation. Leaves inoculated with conidia or sterile water at 6 and 24 h on ‘Seli’ and ‘Cuiguan’ were collected for enzyme activity analysis. We used chemical kits to measure the enzyme activity of POD, PPO, and H_2_O_2_ content according to the manufacturer’s protocol. The assay kit was commercially acquired from Nanjing Jiancheng Bioengineering Institute, Nanjing, China. Each experiment was replicated for three times.

### RNA isolation and sequencing

After inoculation with *C. fructicola* conidia or sterile water at 6 and 24 h, 24 ‘Seli’ and ‘Cuiguan’ samples were collected for RNA-seq. Total RNA was extracted according to the manufacturer’s instructions of HiPure HP Plant RNA Mini Kit (Magen, Guangzhou, China), and RNA libraries were constructed using 1 µg of the total RNA by Wuhan Metwell Biotechnology Co. Ltd. Agilent 2100 Bioanalyzer (Agilent, CA, USA) was used to confirm the RNA quality. The library was sequenced on an Illumina HiSeq 2500 platform, and 150 bp paired-end reads were generated. Clean data were obtained by removing adapters, ploy-N and low quality reads from the raw data. The RNA-seq reads were mapped to the reference genome of ‘Cuiguan’ pear (*P. pyrifolia*) [50] using HISAT [51]. The RNA-seq raw data have been deposited in the NCBI sequencing read archive database under the GenBank accession number PRJNA1078101. Principal component analysis (PCA) was conducted using the prcomp function within the R software (https://www.R-project.org/). The correlation coefficient was calculated using the cor function in the R package, and the correlation heat map was plotted using the corrplot package.

### Identification of DEGs and coexpression network analysis

To identify the DEGs of ‘Seli’ and ‘Cuiguan’ after inoculation, gene expression levels were normalized to reads per kilobase of transcript sequence per million using Cufflinks package [52]. Genes with P-values < 0.05 and |log2 fold change| ≥1 were defined as DEGs. GO and KEGG enrichment pathway analyses were performed using GOATOOLS and KOBAS software.

Weighted gene coexpression network analysis was performed using the WGCNA package [53]. WGCNA was used to construct cluster trees based on the correlations of gene expression levels and different modules depicted with different colors as well as the correlation between the modules and samples. Protein–protein interaction networks were constructed based on the STRING protein interaction database (https://string-db.org/) and visualized using Cytoscape software [54]. The nodes indicated with a brown circle in the brown module of the network represent proteins encoded by the top 30 DEGs.

### qRT–PCR validation

First-strand cDNA was synthesized from 1 µg of RNA using Maxima H Minus First-Strand cDNA Synthesis Kit (Thermo Scientific, Shanghai, China). The primer sequences are listed in Table S3 in Additional File 1. qRT–PCR was performed using three independent repetitions using SYBR Green PCR Master Mix (Applied Biosystems, USA) via the CFX96 Optics Module. The PCR protocol parameters were as follows: 95℃ for 30 s, followed by 40 cycles of 95℃ for 10 s and 60℃ for 30 s. The relative gene expression levels were calculated using the 2^−ΔΔCt^ method, with actin as the reference gene.

### Electronic supplementary material

Below is the link to the electronic supplementary material.

Supplementary Material 1

Supplementary Material 2

## Data Availability

The data presented in this study can be found in the article and the supplementary materials, and the generated raw reads have been uploaded to NCBI with genbank accession numbers of PRJNA1078101.
