# Supplementary material for: Determination of anthracnose (Colletotrichum fructicola) resistance mechanism using transcriptome analysis of resistant and susceptible pear (Pyrus pyrifolia)
Source: BMC Plant Biol. 2024 Jun 28;24:619. doi: 10.1186/s12870-024-05077-6 (PMC11212231; doi:10.1186/s12870-024-05077-6)
Supplement: Supplementary file 1 — Supplementary Material 1 [file 12870_2024_5077_MOESM1_ESM.docx]

**Supporting information**

**
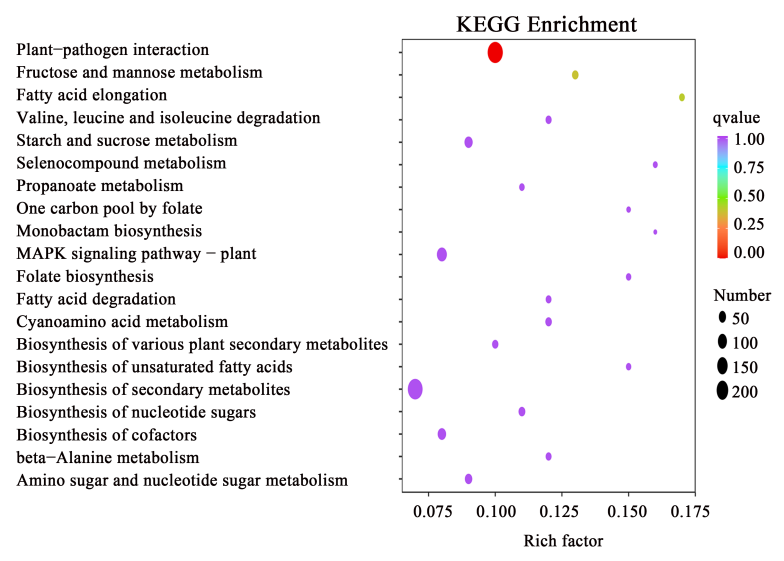
**

**Fig S1.** KEGG enrichment of differentially expressed genes (DEGs) in the brown module following *Colletotrichum fructicola* infection in ‘Seli’ and ‘Cuiguan’ leaves at 6 and 24 h. The q-value is an adjusted p-value ranging from 0 to 1, and a lower value indicates greater pathway enrichment.



**Fig S2.** qRT–PCR validation of the selected differentially expressed genes (DEGs) in ‘Seli’ and ‘Cuiguan’ leaves after *C. fructicola* inoculation. **(a-f)** Nine genes were evaluated: BRI1-associated receptor kinase 1 (*BAK1*), [MAP kinase substrate 1 (*MKS1*](https://www.sciencedirect.com/science/article/pii/S1570963907001550)), polyphenol oxidase (*PPO*), mitogen-activated protein kinase 3 (*MPK3*), WRKY-type transcription factor 29 (*WRKY29*), male discoverer 1-interacting receptor-like kinase 2 (*MIK2*), calmodulin-like protein 19 (*CML19*), calcium-dependent protein kinase (*CDPK*), and cinnamyl alcohol dehydrogenase 5 (*CAD5*). The treatment group was inoculated with *C. fructicola*, and the control group was inoculated with sterile water at 6 and 24 h. Gene expression levels were normalized to respective control group of ‘Seli’ and ‘Cuiguan’ at 6 and 24 h post-inoculation. The relative expression level was calculated using the 2^−ΔΔCt^ method with actin as the reference gene. The statistical analysis was carried out by GraphPad Prism 8 software. Error bars indicate the standard deviation of three independent repetitions.
