# Supplementary material for: Determination of anthracnose (Colletotrichum fructicola) resistance mechanism using transcriptome analysis of resistant and susceptible pear (Pyrus pyrifolia)
Source: BMC Plant Biol. 2024 Jun 28;24:619. doi: 10.1186/s12870-024-05077-6 (PMC11212231; doi:10.1186/s12870-024-05077-6)
Supplement: Supplementary file 2 — Supplementary Material 2 [file 12870_2024_5077_MOESM2_ESM.docx]

**Supporting information**

**Table S1.** Summary of RNA sequencing and mapping using the *Pyrus pyrifolia* genome as the reference.

| Sample | Raw Reads | Clean Reads | Clean Base(G) | Error Rate(%) | Q20(%) | Q30(%) | GC Content(%) |
| --- | --- | --- | --- | --- | --- | --- | --- |
| SL_D6-1 | 49346478 | 47480938 | 7.12 | 0.03 | 97.58 | 93.09 | 45.73 |
| SL_D6-2 | 50439926 | 49126722 | 7.37 | 0.03 | 97.39 | 92.69 | 46.14 |
| SL_D6-3 | 51130736 | 49452214 | 7.42 | 0.03 | 97.67 | 93.32 | 46.39 |
| SL_CF6-1 | 51055534 | 49498224 | 7.42 | 0.03 | 97.6 | 93.17 | 46.09 |
| SL_CF6-2 | 49196598 | 47704632 | 7.16 | 0.03 | 97.53 | 93.01 | 46.13 |
| SL_CF6-3 | 47236250 | 46050914 | 6.91 | 0.03 | 97.29 | 92.46 | 46.08 |
| CG_D6-1 | 50640476 | 48689360 | 7.3 | 0.03 | 97.46 | 92.89 | 45.83 |
| CG_D6-2 | 50757548 | 48512478 | 7.28 | 0.03 | 97.86 | 93.75 | 45.08 |
| CG_D6-3 | 49938926 | 47979026 | 7.2 | 0.03 | 97.77 | 93.44 | 43.27 |
| CG_CF6-1 | 53718672 | 51765722 | 7.76 | 0.03 | 97.7 | 93.37 | 44.99 |
| CG_CF6-2 | 44979476 | 43762712 | 6.56 | 0.03 | 97.59 | 93.12 | 45.7 |
| CG_CF6-3 | 50933008 | 48442660 | 7.27 | 0.03 | 97.72 | 93.45 | 45.06 |
| SL_D24-1 | 56286490 | 54176726 | 8.13 | 0.03 | 97.63 | 93.29 | 45.22 |
| SL_D24-2 | 53880754 | 52299212 | 7.84 | 0.03 | 97.69 | 93.38 | 46.17 |
| SL_D24-3 | 54217248 | 52850858 | 7.93 | 0.03 | 97.61 | 93.18 | 46.3 |
| SL_CF24-1 | 43719980 | 42555826 | 6.38 | 0.03 | 97.65 | 93.31 | 46.5 |
| SL_CF24-2 | 49546488 | 48367112 | 7.26 | 0.03 | 97.52 | 92.99 | 46.47 |
| SL_CF24-3 | 47566246 | 46370220 | 6.96 | 0.03 | 97.51 | 92.98 | 46.72 |
| CG_D24-1 | 47247558 | 46140886 | 6.92 | 0.03 | 97.4 | 92.73 | 45.94 |
| CG_D24-2 | 44201236 | 43127598 | 6.47 | 0.03 | 97.49 | 92.89 | 45.97 |
| CG_D24-3 | 46987228 | 45912186 | 6.89 | 0.03 | 97.57 | 93.11 | 46.04 |
| CG_CF24-1 | 54153182 | 52120198 | 7.82 | 0.03 | 97.63 | 93.2 | 45.23 |
| CG_CF24-2 | 58860966 | 57150358 | 8.57 | 0.03 | 97.72 | 93.43 | 44.78 |
| CG_CF24-3 | 51717636 | 50079610 | 7.51 | 0.03 | 97.54 | 92.96 | 45.16 |

Note: SL_D and CG_D represent resistant variety ‘Seli’ and susceptible cultivar ‘Cuiguan’ inoculation with sterile water at 6 and 24 h. SL_CF and CG_CF indicate that ‘Seli’ and ‘Cuiguan’ inoculation with *Colletotrichum fructicola* at 6 and 24 h.

**Table S2.** List of the eight differentially expressed genes in calcium signaling pathway after *C. fructicola* inoculation in ‘Seli’ and ‘Cuiguan’.

| Term  Target description | Gene ID | Fold change 6 h inoculation  Seli Cuiguan | | Fold change 24 h inoculation  Seli Cuiguan | |
| --- | --- | --- | --- | --- | --- |
| Respiratory burst oxidase homolog protein D-like Calmodulin-like protein 5  Calmodulin-like protein 19  Calmodulin-like protein 19  Calmodulin-like protein 30  Calmodulin-like protein 38  Calmodulin-like protein 25  Calcium-dependent protein kinase2-like | EVM0014939  EVM0024348  EVM0022441  EVM0018291  EVM0003685  EVM0035428  EVM0006104  EVM0034481 | 1.1  1.4  1.4  1.7  -  -  -  - | -  1.1  -  -  -  -  -  - | -  -  -  -  1.8  2.4  1.2  4.1 | -  1.8  -  -  -  -  -  - |

Note: ‘-’ indicates no significant difference.

**Table S3.** qRT-PCR Primer sequences used in this study.

| Primer ID | Sequence of primers (5’-3’) |
| --- | --- |
| MPK3-F | GGCAGACCTCGTTCCCGCTAA |
| MPK3-R | CCACCATCTCCTTCGTCTCCG |
| WRKY29-F(EVM0036956) | CCACCAACTATTCTTGCCTCC |
| WRKY29-R(EVM0036956) | CATACTTACGCCACGCCCACA |
| MKS1-F(EVM0025791) | GATGATAATCGTCCAGCACAA |
| MKS1-R(EVM0025791) | CTCTTCTGATTTATTGGCGGC |
| PPO-F(EVM0001400) | CATCAATCTCGTGCAAAGCCAC |
| PPO-R(EVM0001400) | CGAGGCCAAGTAGGACATTTCTC |
| BAK1-F(EVM0006468) | GCCAGATCATATCTGGAACCATG |
| BAK1-R(EVM0006468) | GATGTTAGAAAGTGTACACGCGG |
| CAD5-F | CAGTTCTGCAAAGAGAGAGGATT |
| CAD5-R | GACGAACCTGTACCTAACGTCG |
| CDPK-F | ATGGGCAAGTGTCTAAGCAAAAG |
| CDPK-R | ACGGTTTTCCGAGGATTGTGTTG |
| CML19-F(EVM0022441） | GGTGATGGAAAGATTTCGCCTTC |
| CML19-R(EVM0022441） | CCCTCCTTCCATTAACCTCAC |
| MIK2-F | CAGATGCCTCCCTTTCTCCTTCA |
| MIK2-R  Actin-F  Actin-R | GTTTTGGTATTGCGGTGACAGAC  TGGTGTCATGGTTGGTATGG  CAGGAGCAACACGAAGTTCA |
